# Universal Platform Based on Carbon Nanotubes Functionalised with Carboxylic Acid Groups for Multi-Analyte Enzymatic Biosensing

**DOI:** 10.3390/bios15100686

**Published:** 2025-10-10

**Authors:** Edmundas Lukoševičius, Julija Kravčenko, Grėta Mikėnaitė, Augustas Markevičius, Gintautas Bagdžiūnas

**Affiliations:** Group of Supramolecular Analysis and Bioelectronics, Institute of Biochemistry at Life Sciences Centre, Vilnius University, Saulėtekio av. 7, LT-10257 Vilnius, Lithuania

**Keywords:** carbon nanotube, oxygen-sensitive electrode, enzymatic biosensor, electrochemical platform

## Abstract

This work presents the development of carbon nanotubes functionalised with carboxylic acid groups (CNT-COOH) as an oxygen-sensitive electrochemical platform for parallel multi-analyte enzymatic biosensing. The platform was constructed by depositing carboxylic-acid-functionalised single-walled carbon nanotubes covalently onto nanostructured gold electrodes modified with a self-assembled monolayer of 4-aminothiophenol. Atomic force microscopy characterization revealed that the nanotubes attached via their ends to the surface and had a predominantly horizontal orientation. Glucose oxidase, lactate oxidase, glutamate oxidase, and tyrosinase were immobilised onto the electrodes to create selective biosensor for lactate, glucose, glutamate, and dopamine, respectively. A key finding is that incorporating catalase significantly extends the linear detection range for analytes by mitigating the accumulation of hydrogen peroxide. The resulting multifunctional biosensor demonstrated its capability for the simultaneous and independent measurement of glucose, lactate as the key bioanalytes under uniform conditions in blood plasma samples, highlighting its potential for applications in health and food technologies.

## 1. Introduction

Carbon materials, including carbon nanotubes (CNTs), graphene, and fullerenes, have significant applications as nanostructured elements and electron donors in bioelectrodes to monitor electrophysiological and biochemical signals [1]. Among these, CNTs possess unique electrochemical properties that render them highly attractive for biomedical applications [2]. Consequently, CNTs have become one of the most widely utilised nanomaterials in this field. Their unique structure and nanoscale dimensions, which include an extensive surface area to volume ratio and high aspect ratio, coupled with excellent electrical conductivity, bestow upon CNTs many beneficial properties for chemical and biosensing applications [3]. The quick progress of multimodal biosensors is motivated by innovations in nanotechnology and the development of novel materials that enhance their performance, durability, and adaptability [4].

Biosensors are revolutionising various fields due to their ability to accurately and rapidly detect the presence and concentration of diverse bioanalytes. This capability supplements societal health, particularly food processing, safety, and technology. For example, rapid glucose monitoring is essential for managing diabetes and controlling added glucose in food [5]. Similarly, lactate levels serve as vital indicators: in blood and sweat, they signal fatigue, while in foods, their presence indicates fermentation and spoilage [6]. To put these into perspective, the normal glucose concentration in blood plasma is approximately five mM, and resting blood lactate in healthy individuals typically ranges from 0.5 to 1 mM. Beyond these, the detection of monosodium glutamate (L-glutamate), a familiar flavour enhancer (E621), is crucial in food analysis [7]. In health, glutamate and dopamine are key neurotransmitters in the central nervous system that regulate mood. Elevated levels of these neurotransmitters can be indicative of mental illness [8]. While the concentrations of glutamate in plasma are 0.050–0.100 mM and in the whole brain are 10–12 mM [9], the concentration of dopamine in the brain is remarkably lower, at only 0.5–5 nM [10]. The vast difference in these concentration ranges makes the simultaneous measurement of multiple analytes a significant analytical challenge.

Traditional multifunctional detection techniques, such as chromatography, often face limitations including high cost, lack of on-site detection capabilities, and the need for skilled operators. In contrast, electrochemical enzymatic biosensors offer a compelling alternative. They have gained increasing attention due to their efficiency in measuring bioanalyte levels. Consequently, developing biosensors capable of simultaneously detecting multiple bioanalytes represents a critical and contemporary challenge with broad implications for health and food technologies [11].

On the one hand, parallel multi-analyte measurement refers to the capability of a biosensor to detect and measure multiple types of signals originating from different biochemical reactions [12]. On the other hand, electrochemical biosensors commonly utilise a three-electrode system. This system can consist of multiple working electrodes, each designed to recognise a specific target analyte, a counter electrode that acts as the current source, and a reference electrode to maintain a stable potential. Electrochemical biosensors are widely employed for multi-analyte measurements because they incorporate multiple working electrodes as the sensing elements, with each electrode dedicated to simultaneously measuring a distinct signal [13]. Enzyme-based multifunctional bioelectrodes must adhere to strict requirements: compatibility with the enzymes used and registering a signal at a specific potential for each analyte [14]. The use of a single type of transducer material for all electrodes, and operation within a single medium (i.e., a buffer of uniform composition), since all measurements must be performed simultaneously in the same electrochemical cell.

Highlighting the biorecognition elements is crucial when discussing biosensors. Oxidase enzymes, classified as oxidoreductases, utilise dioxygen as their electron acceptor. These enzymes are frequently employed as selective recognition elements in enzymatic electrochemical biosensors, which are key to successful application and commercialisation [15]. Typically, these enzymes use oxygen to regenerate their flavin adenine dinucleotide or copper complex-based cofactors after oxidising the substrate. Electrochemical biosensors based on enzymatic reactions are generally categorised into three generations [16]. First-generation biosensors quantify the concentration of substrates or products, such as hydrogen peroxide, or measure the decrease in oxygen concentration resulting from the enzymatic reaction [17]. Second-generation biosensors employ a redox mediator to facilitate electron transfer between the enzyme (i.e., oxidase) cofactor and the electrode surface. Conversely, third-generation biosensors achieve direct electron (or hole) transfer between the oxidase and the electrode, eliminating the need for a mediator [18]. Consequently, employing oxygen-sensitive electrodes is particularly convenient for multifunctional biosensors utilising oxidase enzymes, as oxygen is inherently involved in the enzymatic reactions occurring at the bioelectrode surface.

A central aspect of biosensor detection is signal transduction coupled with the selective recognition of the target biological species. A common strategy to enhance biosensor sensitivity involves functionalising carbon nanotubes (CNTs) by covalently binding them with enzymes [19]. For instance, the electrocatalytic properties of CNTs can enhance the electroactivity of enzymes and facilitate the electron-transfer reactions through CNT to/from the electrode [20]. However, these covalent functionalisation methods often lead to partial enzyme denaturation or are overly complex and may not consistently yield the desired improvements. Consequently, the oxygen reduction reaction (ORR), a prominent action mechanism of CNTs at the cathode [21], plays a crucial role in the overall performance of fuel cells, metal-air batteries and enzymatic biosensors.

In this work, we developed a universal oxygen-sensitive platform for enzymatic electrochemical biosensors based on carbon nanotubes functionalised with carboxylic acid groups and deposited onto gold nanoparticles. Lactate oxidase, glucose oxidase, L-glutamate oxidase, and tyrosinase were immobilised onto this platform to create stable and selective bioelectrodes for the determination of glucose, lactate, glutamate, and dopamine, respectively. We have demonstrated the applicability of this platform for the parallel measurement of the concentrations of these analytes under uniform conditions in real-world samples.

## 2. Materials and Methods

### 2.1. Materials

Oxidases such as L-lactate oxidase (LOx from *Aerococcus viridans* species, 40 U mg^−1^, Sigma-Aldrich, St. Louis, MO, USA), L-glutamate oxidase (LGOx from *Streptomyces* sp., >5 U mg^−1^, Sigma-Aldrich), catalase (CAT from *Aspergillus niger*, ammonium sulfate suspension, >4000 U mg^−1^ protein, Sigma-Aldrich), glucose oxidase (GOx from *Aspergillus niger*, >100,000 U mg^−1^ protein, Sigma-Aldrich), and tyrosinase (TYR, from mushrooms, >1000 U mg^−1^ protein, Sigma-Aldrich) were used. Carboxylic-acid-functionalised single-wall carbon nanotubes (CNT-COOH, D × L 4–5 nm × 0.5–1.5 μm, Sigma-Aldrich), sodium L-lactate (Lac, 99%, Sigma-Aldrich), D-glucose (99%, Sigma-Aldrich), 4-aminothiophenol (ATP, 97%, Sigma-Aldrich), dialysis membrane Membra-CelTM (MD25 14 × 500 CLR, Carl Roth, Karlsruhe, Germany), 1,1′-carbonyldiimidazole (CDI, ≥90.0%, Alfa Aesar, Haverhill, MA, USA), *N*,*N*-dimethylformamide (DMF, anhydrous, 99.8%, Sigma-Aldrich), *N*,*N*-diisopropylethylamine (DIPEA, ≥90.0%, Sigma-Aldrich), methanol (≥99.9%, Sigma-Aldrich) were used. Commercial DMF was stored on 4 Å molecular sieves to obtain the anhydrous solvent in a refrigerator in the dark. The 50 mM potassium phosphate buffer (PPB) solution of pH 7.0 used in this work contained an additional 100 mM KCl. PPB 50 mM without KCl was employed to prepare enzyme solutions. pH of the PPB was adjusted to pH 7.0 with KOH or HCl solution. All the chemicals were analytical-grade reagents, used without further purification, and prepared by dilution using deionised water or PPB. Gold nanoparticles (AuNP) of a diameter of 20 nm were prepared using the Turkevich method, incorporating the size-control modifications described by Sivaraman et al. [21]. The food products were purchased from the nearest store.

### 2.2. Electrochemical Instrumentation

All the cyclic voltammetry (CV) and chronoamperometry (CA) measurements were performed using the PalmSens4 potentiostat (PalmSens, Houten, The Netherlands). For the experiments with two working electrodes, the PalmSens4 was expanded with a BiPot module for use with a second working electrode. Data was analysed with a PSTrace program (version 5.9). Gold (Au) working electrodes (0.031 cm^2^), Ag/AgCl with saturated solution KCl reference electrode from PalmSens and a titanium plate (~0.5 cm^2^) as the counter electrode were used for our electrochemical measurements. The density of current was calculated by dividing the current by 0.031 cm^2^, the geometric area of the Au electrodes. An electrochemical cell of 10 mL was used for the electrode studies. To analyse real samples, we used a small electrochemical cell containing 3.0 mL of PPB solution (pH 7.0), to which 160 µL of HN and 80 µL of HP real samples were added under stirring. The measurements were repeated two times (*n* = 2).

### 2.3. Construction of the Au/AuNP/CNT-COOH Bioelectrodes

The working Au electrodes were mechanically polished using aluminium paste (0.3 μm) in circular motion and sonicated in distilled water for 5 min. Electrochemical cleaning was performed using CV in a potential range from −2.6 V to 0 V in 50 mM KOH at a 300 mV s^−1^ scan rate for 40 cycles, then electrochemically cycled in 0.5 M H_2_SO_4_ solution in a range from −0.2 V to 1.75 V at 300 mV s^−1^ for 40 cycles. 3 μL of AuNP were put on the clean Au electrode and left to dry, and the electrode was electrochemically cycled with cyclic voltammetry in 0.5 M H_2_SO_4_ solution at 300 mV s^−1^ for 20 cycles. For the functionalisation of amino groups onto AuNP, ATP was dissolved in methanol, and 100 μL was used to prepare the 5 mM solution. The Au electrodes with the AuNP tops were submerged in an ATP (5 mM) solution and left at +4 °C temperature for 12 h to create a self-assembly monolayer (SAM). 1 mg of CNT-COOH was dispersed in a 100 μL DMF and 5 μL DIPEA mixture. Then, this mixture was sonicated for 30 min. The Au/AuNP/ATP electrodes were washed with distilled water and left to dry. Next, 2 mg of CDI was added to the mixture and vortexed for 2 min. 3 μL of this mixture was dripped on the surface of the dry gold electrodes and left for 45 min under the fume hood.

To immobilise enzymes onto the electrodes, all the prepared Au/AuNP/ATP/CNT-COOH electrodes were washed with distilled water and dried. Then, the corresponding enzyme stock solution was diluted to 1 mg mL^−1^ with 50 mM PPB at pH 7.0. Then, 3 μL of the enzyme was dripped on the prepared electrode surface and left to partially dry. To immobilise the corresponding enzyme and the CAT mixture onto the prepared electrodes, all the prepared electrodes were washed with distilled water and left for 5 min. First, 1 μL of a solution of CAT (20 mg mL^−1^) was mixed with 20 μL of a solution of the enzyme. 3 μL of this mixture was dripped on the prepared electrode and left to partially dry. The dialysis membrane was cut into smaller pieces (4 cm^2^) and washed with distilled water to apply the dialysis membrane onto the prepared electrode. These washed, smaller pieces of dialysis membrane were applied onto the prepared electrode with the immobilised enzyme.

### 2.4. Electrochemical Measurements

Chronoamperometry (CA) method was used for the titration, repeatability and selectivity measurements. For the titration and repeatability, the solutions of sodium lactate (100 mM), glutamate (100 mM), glucose (1.00 M), and dopamine (10.0 mM) were prepared in PPB pH 7.0 with 100 mM KCl. The titration curves were plotted for the concentrations ranging from 5 µM to 10 mM, and it was measured at −0.20 V versus Ag/AgCl. The repeatability was measured at the same voltage by adding a solution of the corresponding analyte three times. For selectivity, the solution of the corresponding analyte was added to the cell, and later, different possible interferences found in mammal blood, such as neurotransmitters and metabolites, were added. After that, a solution for the corresponding analyte was added to check if the bioelectrode was still working. All these measurements were carried out at room temperature.

The sensitivities of the electrodes were calculated from the slope (*S*) derived from the linear concentration range of the respective titration. These sensitivities were then normalised by the geometric area of the electrodes (diameter: 0.20 cm; area: 0.031 cm^2^). The limit of detection (LOD), defined as the lowest concentration of a substance that can be reliably detected, was calculated using the LOD = 3.0 *σ*/*S* equation, where *σ* is the standard deviation of the obtained results and *S* is the slope of the calibration curve.

### 2.5. AFM Measurements

The morphology of the prepared surfaces was analysed by Atomic Force Microscopy (AFM) using a Dimension Icon AFM system (Bruker, Billerica, MA, USA), operated in tapping mode in air. For these studies, gold surfaces were fabricated by evaporating gold onto a smooth glass surface. All layers, except AuNPs, were immobilised on these surfaces for AFM characterisation.

## 3. Results

### 3.1. Molecular Oxygen-Sensitive Electrode Preparation

**Figure 1:**
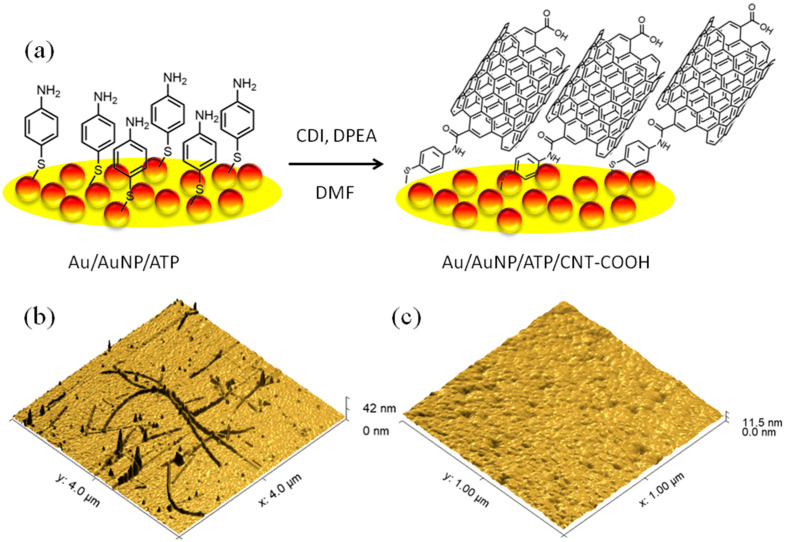
(**a**) Schematic representation of the Au/AuNP/CNT-COOH electrode fabrication and the amide coupling reaction; (**b**) AFM image (4 µm × 4 µm) visualising the covalently immobilised CNT-COOH on the Au surface; (**c**) AFM image (1 µm × 1 µm) of the bare Au surface.

Carboxylic acid-functionalised single-walled carbon nanotubes (CNT-COOH) were deposited onto a nanostructured gold (Au) electrode to fabricate the oxygen-sensitive electrode. Initially, the bare Au electrode was meticulously cleaned mechanically and electrochemically using reductive and oxidative conditions. Subsequently, gold nanoparticles (AuNPs) with a diameter of 20 nm were deposited onto the cleaned Au electrodes. These AuNP-modified electrodes were then electrochemically cleaned under oxidative conditions to obtain a pristine Au/AuNP electrode surface. Following the AuNP deposition, the electrochemical surface area of the Au/AuNP electrode was determined. Based on a comparison of the cathodic currents at a potential of +0.82 V versus Ag/AgCl in 3 M KCl (all potentials reported herein are referenced against this electrode) in 0.5 M H_2_SO_4_ (Figure S1), the Au/AuNP electrode exhibited a surface area 16 times larger than that of the bare Au electrode (a detailed description of the calculation methodology is presented in the Supporting Information). Next, a self-assembled monolayer (SAM) of 4-aminothiophenol (ATP) was immobilised onto the electrochemically cleaned Au/AuNP electrode, creating an amino group-functionalised surface. Finally, the CNT-COOH molecules were chemically deposited onto this amino-functionalised surface via amide bond formation. This reaction utilised 1,1′-carbonyldiimidazole (CDI) as the coupling reagent and *N*,*N*-diisopropylethylamine (DIPEA) as the base in anhydrous *N*,*N*-dimethylformamide (DMF). Ensuring the DMF solvent is both anhydrous and devoid of *N*,*N*-dimethylamine impurities is critical, as these impurities can competitively react with the amino groups of the SAM during the coupling reaction. The resulting Au/AuNP/CNT-COOH electrode was used in subsequent research. These procedures are illustrated in Figure 1a.

### 3.2. Characterisation of the CNT-COOH on the Au Surface

The CNT-COOH nanotubes was covalently immobilised onto the gold surface, which was previously modified with an ATP monolayer. We removed any excess CNT-COOH by thoroughly washing the surface with DMF, water, and methanol. This covalent attachment, facilitated by the carboxylic acid groups, ensured that only the functionalised CNTs were bound to the Au surface. We characterised the resulting surface using Atomic Force Microscopy (AFM). Figure 1b,c displays the AFM images of the surfaces of covalently immobilised CNT-COOH and bare gold, respectively.

The lengths of the CNT-COOH structures ranged from 200 nm to 1.5 µm. We measured the average height and diameter of these structures to be 3.4 nm and 48 nm, respectively. It is well-established that the carboxylic acid functionalisation of carbon nanotubes primarily occurs at their ends [22]. Based on our experimental results, the nanotubes were covalently attached to the surface via their ends and exhibited a predominantly horizontal orientation. To increase the surface loading of CNT-COOH, we immobilised them on gold nanoparticles (AuNPs). However, due to its significant roughness, we did not perform the AFM characterisation of this AuNP-modified surface.

### 3.3. Sensitivity to Molecular Oxygen of the Electrodes

We electrochemically tested the prepared Au/AuNP and Au/AuNP/CNT-COOH electrodes using cyclic voltammetry (CV). These experiments were conducted first with the natural concentration of molecular oxygen in the buffer solution, and then after removing molecular oxygen by bubbling argon through the solution. As Figure S2 illustrates, the Au/AuNP/CNT-COOH electrode is sensitive to the concentration of molecular oxygen. The cathodic currents at −0.20 V increased from −4.2 µA to −1.7 µA when molecular oxygen was present versus absent in the electrolyte. This sensitivity indicates that this electrode can be applied as a first-generation biosensor component sensitive to molecular oxygen.

In contrast, as shown in Figure S2, a gold electrode coated with AuNPs and an ATP monolayer (Au/AuNP/ATP) does not exhibit these oxygen-sensitive properties. To further evaluate the biosensing capabilities, we applied a solution of lactate oxidase (LOx, 1 mg mL^−1^) to the Au/AuNP/CNT-COOH electrode and allowed it to dry. The enzyme was immobilised on the electrode surface using a dialysis membrane impermeable to biomolecules larger than 14 kDa. The resulting Au/AuNP/CNT-COOH/LOx electrode was titrated with lactate concentrations ranging from 5 µM to 3.4 mM, using a constant potential of −0.20 V. The sensitivity and limit of detection (LOD) for lactate were calculated to be 12 µA mM^−1^ cm^−2^ and 15 µM, respectively, for the Au/AuNP/CNT-COOH/LOx bioelectrode (Table 1, entry 1). It is worth noting that the Au/CNT-COOH/LOx electrode without nanoparticles showed a low response to the analyte. This suggests that the primary role of the immobilized nanotubes is the enhancement of molecular oxygen sensitivity, rather than a significant increase in the effective surface area of the electrode.

**Figure 2:**
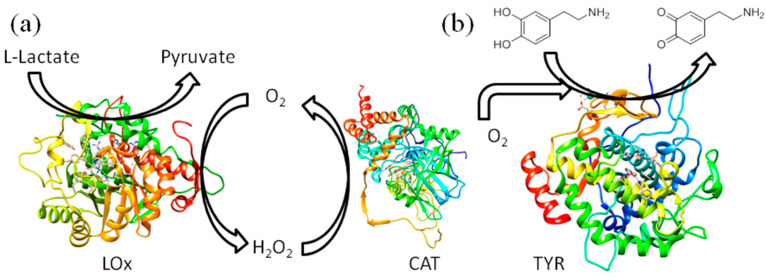
Enzymatic reactions on the bioelectrodes for the detection of (**a**) lactate and (**b**) dopamine.

Previous research by Berketa et al. [23] has demonstrated that the analytical characteristics of oxidase-based biosensors can be improved by incorporating catalase (CAT). Furthermore, Valdes and Moussy [24] found that enzymes deactivated over time, primarily due to oxidation by hydrogen peroxide. Therefore, to enhance the biocatalytic properties of this bioelectrode, we prepared a mixture of the LOx enzyme with CAT. CAT catalyses the conversion of the resulting hydrogen peroxide into molecular oxygen (Figure 2a), thereby increasing the molecular oxygen concentration in the reaction mixture on the electrode surface. For the new Au/AuNP/CNT-COOH/LOx+CAT bioelectrode, the sensitivity and LOD were estimated to be 7.5 µA mM^−1^ cm^−2^ and 66 µM, respectively (Table 1, entry 2). The repeatability, assessed by the relative standard deviation (RSD) of the current response after adding 0.20 mM of lactate (*n* = 3), was found to be similar for both the Au/AuNP/CNT-COOH/LOx and Au/AuNP/CNT-COOH/LOx+CAT bioelectrodes, at 5.2% and 5.6%, respectively. Although the Au/AuNP/CNT-COOH/LOx+CAT bioelectrode exhibited somewhat lower sensitivity and a higher LOD, the inclusion of CAT resulted in a wider linearity range. This broader linear range is a significant advantage when analysing real samples, as it extends the detection capabilities (Table 1, entries 1 and 2). This observation applies to all the bioelectrodes with flavin adenine dinucleotide (FAD)-based oxidases we have studied. Additionally, incorporating CAT had almost no effect on the response time (Table 1). A summary of recent enzymatic biosensors for chronoamperometric sensing of lactate indicates that sensitivities typically range from 0.02 to 41 µA mM^−1^ cm^−2^ and LODs from a few tens to several hundreds of µM [6]. Consequently, our biosensor exhibits comparable characteristics and is suitable for determining lactate concentrations in real samples.

### 3.4. Performance of the Bioelectrodes

**Figure 3:**
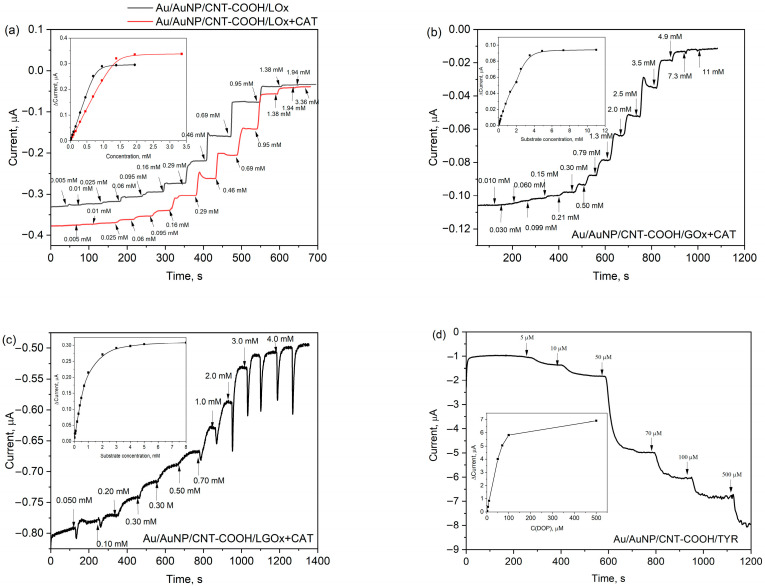
The response to the corresponding substrate of the bioelectrodes at −0.20 V in PPB pH 7.0: (**a**) Au/AuNP/CNT-COOH/LOx electrode without and with CAT; (**b**) Au/AuNP/CNT-COOH/GOx+CAT; (**c**) Au/AuNP/CNT-COOH/LGOx+CAT; (**d**) Au/AuNP/CNT-COOH/TYR. The insets show the current responses to the concentration of corresponding substrates.

The Au/AuNP/CNT-COOH electrode was used to selectively determine lactate, glucose, glutamate, and dopamine. Glucose oxidase (GOx) from Aspergillus niger, L-glutamate oxidase (LGOx) from Streptomyces sp. (with additive CAT from Aspergillus niger), and tyrosinase (TYR) from mushrooms served as the biorecognition elements for these respective bioelectrodes. All our bioelectrodes performed effectively at a potential of −0.20 V in a 50 mM PPB (pH 7.0) containing 100 mM KCl. The chronoamperometric curves obtained at −0.20 V during the titration of lactate (Figure 3a), glucose (Figure 3b), glutamate (Figure 3c), and dopamine (Figure 3d) are presented. The response times (defined as the time from analyte addition to current stabilisation) for the Au/CNT-COOH/LOx, GOx, LGOx+CAT, and TYR bioelectrodes are summarized in Table 1. The performance results for these Au/AuNP/enzyme-based bioelectrodes, which include LOx (both with and without CAT), GOx, LGOx with CAT, and TYR, are also presented in Table 1.

The Au/AuNP/CNT-COOH/LGOx+CAT bioelectrode exhibited a sensitivity of 6.9 µA mM^−1^ cm^−2^, a linear range from 0.010 mM to 1.0 mM, and a LOD of 130 µM. Similarly, the sensitivity, linearity, and LOD as the characteristics for the Au/AuNP/CNT-COOH/GOx+CAT bioelectrode were determined to be 0.88 µA mM^−1^ cm^−2^, from 0.20 mM to 2.5 mM, and LOD of 250 µM, respectively. These bioelectrodes demonstrated characteristics comparable to those reported in the literature [5,7], confirming their suitability for determining glutamate and glucose concentrations, respectively. CAT was not added for the Au/AuNP/CNT-COOH/TYR bioelectrode because hydrogen peroxide is not a byproduct of its enzymatic reaction. Interestingly, we observed a decrease in cathodic currents in this case, suggesting a different mechanism of action. We propose that the TYR enzyme catalyses the oxidation of dopamine to its quinone form in the presence of molecular oxygen. This quinone form is then reduced on the nanostructured electrode surface (Figure 2b). Compared to our other bioelectrodes, the Au/AuNP/CNT-COOH/TYR bioelectrode is exceptionally sensitive, showing 2400 µA mM^−1^ cm^−2^, with a low LOD of 6.4 µM. Other electrochemical TYR-based biosensors in the literature demonstrate similar characteristics [25].

### 3.5. Selectivity Assessment

We assessed the selectivity of these bioelectrodes using compounds commonly found in blood samples or food products, including lactate, pyruvate, glucose, ascorbic acid, uric acid, urea, and various L- and D-amino acids. Our experimental protocol involved first adding a solution of the analyte to the electrochemical cell at −0.20 V, followed by solutions of the interfering compounds. Finally, an additional analyte solution was introduced at the end of the chronoamperometry experiment to observe any overall enzyme inhibition. For the Au/AuNP/CNT-COOH/LOx+CAT bioelectrode, we observed no significant changes in current upon adding these interfering substances (Figure S3). Similar experiments were conducted with our other bioelectrodes. The Au/AuNP/CNT-COOH/GOx+CAT electrode showed no interference from common blood reagents or fructose, sucrose, or lactose, which are common carbohydrates in food (Figure S4). We noted low-intensity responses to uric acid for the Au/AuNP/CNT-COOH/LGOx+CAT electrode (Figure S5a). We also tested the most common L-amino acids (Asn, Met, Lys, Thr, Gln, His, Ile, Ser, Pro, Val, Phe) with this bioelectrode (Figure S5b). A response to L-Gln was observed, along with reversible inhibition of the enzyme by adding the same concentration of D-Glu. The Au/AuNP/CNT-COOH/TYR electrode was evaluated using common interferences and aromatic L-amino acids (Tyr and Trp) (Figure S6a), as well as common neurotransmitters [26] such as serotonin, noradrenaline, adrenaline, acetylcholine, histamine, γ-aminobutyric acid (GABA), and melatonin (Figure S6b). We observed a five times lower response to L-tyrosine compared to dopamine, and ten times lower intensity responses to adrenaline, noradrenaline, and serotonin at a concentration of 50 µM. To confirm that the immobilised enzyme solely generated the analytical response of the bioelectrodes, we also tested the Au/AuNP/CNT-COOH electrode without immobilised enzymes (Figure S7). No response to the interferences was observed.

### 3.6. Repeatability and Stability

The repeatability of the bioelectrodes, estimated as the relative standard deviation (RSD), ranged from 3.8% to 6.8% (Table 1). These values are considered suitable for the analysis of these analytes. However, the Au/AuNP/CNT-COOH/TYR bioelectrode initially exhibited poor repeatability and stability (RSD = 12%). To address this, we changed the buffer medium to a 50 mM, pH 7.0 tris(hydroxymethyl)aminomethane (TRIS) buffered saline solution. This buffer modification stabilised the bioelectrode by preventing the reaction of phosphate ions with the copper ion-based cofactor in the active site of TYR, which can form insoluble copper(II) phosphate. Unfortunately, this TRIS buffer is unsuitable for our other tested bioelectrodes or requires further investigation. Moreover, the fabricated bioelectrodes were stored in a refrigerator (+4 °C) and soaked in the PPB for one week. However, the electrodes retained approximately 60% of their initial activity after this period. It suggests these bioelectrodes are primarily intended for single-use applications or multiple measurements within the same day. Additionally, the bioelectrodes must be calibrated daily.

### 3.7. Investigation of Real Samples

To assess the reliability of our glucose biosensors, we utilised Cormay HN (normal) and HP (pathological) bovine blood serums as real samples. Since our electrodes are optimised to operate at the same potential and solution pH, they can be adapted to measure two analytes simultaneously. For these blood serums, we chose to measure glucose and lactate. The manufacturer states that the concentration ranges for glucose and lactate are 4.13–5.05 mM and 3.19–4.97 mM for HN serum, respectively, and 13.3–16.3 mM and 1.06–1.36 mM for HP serum. The optimal potential of −0.20 V was applied using two Au/AuNP/CNT-COOH/LOx+CAT and Au/AuNP/CNT-COOH/GOx+CAT working bioelectrodes in a 3 mL electrochemical cell (Figure S8). A three-point calibration curve was used for the corresponding analyte. Figure S9 shows chronoamperometry curves by adding real samples. The concentrations of glucose and lactate in the HN serum were calculated to be 3.80 ± 0.31 mM and 3.14 ± 0.33 mM, respectively. In the HP serum, the 16.8 ± 1.4 mM concentration of glucose and 1.61 ± 0.35 mM of lactate were estimated. These measured concentrations fall within the confidence intervals of the manufacturer’s declared concentrations for both analytes. The biosensor effectively differentiates between normal and pathological samples primarily due to the observed distinct glucose concentration ranges.

For the glutamate measurements in food products and the HN blood serum, the Au/AuNP/CNT-COOH/LGOx+CAT electrode was employed. The measurements were repeated three times (*n* = 3), utilising the calibration curve and the measured current changes corresponding to specific glutamate concentrations. We determined a glutamate concentration of 1.0 ± 0.2 mM in the HN serum, although the manufacturer does not declare glutamate in these serums. We spiked the HN serum with 5.0 mM glutamate to verify this finding. Subsequent measurement yielded a 6.1 ± 0.4 mM concentration in the spiked sample. Subtracting the added glutamate confirmed a concentration of around 1 mM glutamate in the original serum. We also tested several other food products. Caesar salad dressing (“Felix”) contained 0.15% (mass) glutamate. Recalculating based on dilutions, this corresponded to approximately 1.5 mg of glutamate per 1 g of sauce. Sushi soy sauce (“AJI”) contained approximately 0.35% glutamate, or about 3.5 mg per 1 g of sauce. Approximately 10% mass glutamate was measured in the “Vegeta” bouillon cube, equating to about 0.1 g of glutamate per 1 g of the bouillon cube. In “Kania” ketchup, the glutamate concentration was 0.39%, or about 3.9 mg per 1 g. These results demonstrate that the Au/AuNP/CNT-COOH/LGOx+CAT electrode is well-suited for measuring glutamate concentrations in blood serum and various food products. Due to its insufficient stability, the Au/AuNP/CNT-COOH/TYR electrode was not used to study real samples. Furthermore, the dopamine concentrations in real samples (such as brain lysates) are typically in the nM range. In contrast, the LOD of our bioelectrode is considerably higher at 6.4 µM, making it unsuitable for such low-concentration detection of dopamine.

## 4. Conclusions

We developed novel bioelectrodes featuring covalently attached carboxylic acid-functionalised carbon nanotubes (CNT-COOH) on gold nanoparticle (AuNP) decorated gold electrodes. Our experimental results indicate that these nanotubes are covalently anchored to the surface via their ends and primarily adopt a horizontal orientation. This oxygen-sensitive electrochemical platform proved highly versatile. We immobilised various oxidases, including L-lactate oxidase, D-glucose oxidase, L-glutamate oxidase, and tyrosinase, and employed them as electrochemical biosensors to selectively determine their corresponding analytes. We demonstrated that adding catalase (CAT) to the detection layer significantly extends the linear range of the bioelectrode by degrading the generated hydrogen peroxide into molecular oxygen. Our platform is optimised to operate consistently at a potential of −0.20 V versus Ag/AgCl and a solution pH 7.0. This uniform operating condition allowed us to reliably measure glucose and lactate simultaneously in blood serum samples. Furthermore, our developed bioelectrodes were successfully used to quantify glutamate concentrations in blood serum and various food products. While the tyrosinase-based bioelectrode showed promising analytical characteristics, its insufficient stability unfortunately prevented its application in real sample analysis. In summary, this universal nanostructured gold and carbon nanotube-based biosensing platform can be broadly applied for the selective detection of various analytes, particularly those whose oxidation can be catalysed by flavin adenine dinucleotide (FAD)-based oxidases.

## Figures and Tables

**Table 1:** Characteristics of our electrodes based on CNT-COOH and corresponding enzymes.

Entry	Bioelectrode	Sensitivity, μA mM^−1^ cm^−2^	Linear Range, μM	LOD, μM	Repeatability (RSD) ^a^, %	Response time ^a^, s
1	Au/AuNP/CNT-COOH/LOx	12	10–690	15	5.2	30
2	Au/AuNP/CNT-COOH/LOx+CAT	7.5	10–1380	66	5.6	32
3	Au/AuNP/CNT-COOH/GOx+CAT	0.88	200–2500	250	6.8	52
4	Au/AuNP/CNT-COOH/LGOx+CAT	6.9	20–1000	130	3.8	72
5	Au/AuNP/CNT-COOH/TYR	2400	10–100	6.4	12	86

^a^ Repeatability and response time were tested by repeating the biosensor operation 3 times using 0.20 mM of lactate, 1.0 mM of glucose, 1.0 mM of glutamate, and 50 μM of dopamine, respectively.

## Data Availability

Data is contained within the article or Supplementary Materials.

## Supplementary Materials

The following supporting information can be downloaded at: https://www.mdpi.com/article/10.3390/bios15100686/s1, Figure S1. Cyclic voltammetry (CV) experiment during the electrochemical cleaning of a bare gold electrode; Figure S2. Cyclic voltammetry (CV) experiment of Au/AuNP/CNT-COOH with and without ambient molecular oxygen; Figure S3. Test of interferences of the Au/AuNP/CNT-COOH/LOx+CAT electrode using chronoamperometry; Figure S4. Test of interferences of the Au/AuNP/CNT-COOH/GOx+CAT electrode using chronoamperometry; Figure S5. Test of interferences of the Au/AuNP/CNT-COOH/LGOx+CAT electrode using chronoamperometry; Figure S6. Test of interferences of the Au/AuNP/CNT-COOH/TYR bioelectrode using chronoamperometry; Figure S7. Test of interferences of the Au/AuNP/CNT-COOH electrode using chronoamperometry; Figure S8. Electrochemical cell for the analysis of real samples; Figure S9. Chronoamperometry curves during the analysis of glucose, lactate in the HN and HP serums.

## Abbreviations

The following abbreviations are used in this manuscript:

CNT-COOH	Carbon nanotubes functionalised with carboxylic acid groups
CNTs	Carbon nanotubes
LOx	Lactate oxidase
GOx	Glucose oxidas
CAT	Catalase
LGOx	L-Glutamate oxidase
TYR	Tyrosinase
Lac	L-Lactate
ATP	4-Aminothiophenol
CDI	1,1′-Carbonyldiimidazole
DMF	Dimethylformamide
DIPEA	*N*,*N*-Diisopropylethylamine
PPB	Potassium phosphate buffer
AuNp	Gold nanoparticles
CV	Cyclic voltamperometry
CA	Chronoamperometry
Au	Gold electrode
SAM	Self-assembly monolayer
S	Slope derived from the linear concentration range
LOD	Limit of detection
AFM	Atomic force microscopy
RSD	Relative standard deviation
GABA	γ-Aminobutyric acid
TRIS	Tris(hydroxymethyl)aminomethane
FAD	Flavin adenine dinucleotide
