# Supplementary material for: Universal Platform Based on Carbon Nanotubes Functionalised with Carboxylic Acid Groups for Multi-Analyte Enzymatic Biosensing
Source: Biosensors (Basel). 2025 Oct 10;15(10):686. doi: 10.3390/bios15100686 (PMC12563482; doi:10.3390/bios15100686)
Supplement: Supplementary file 1 [file biosensors-15-00686-s001.zip › biosensors-3914912-supplementary.pdf]

Supporting Information for
Universal Platform based on Carbon Nanotubes Functionalised with Carboxylic Acid Groups for Multi-Analyte Enzymatic Biosensing

*Edmundas Lukoševičius, Julija Kravčenko, Grėta Mikėnaitė, Augustas Markevičius, Gintautas Bagdžiūnas**

Group of Supramolecular Analysis and Bioelectronics, Institute of Biochemistry at Life Sciences Centre, Vilnius University, Saulėtekio av. 7, LT-10257 Vilnius, Lithuania

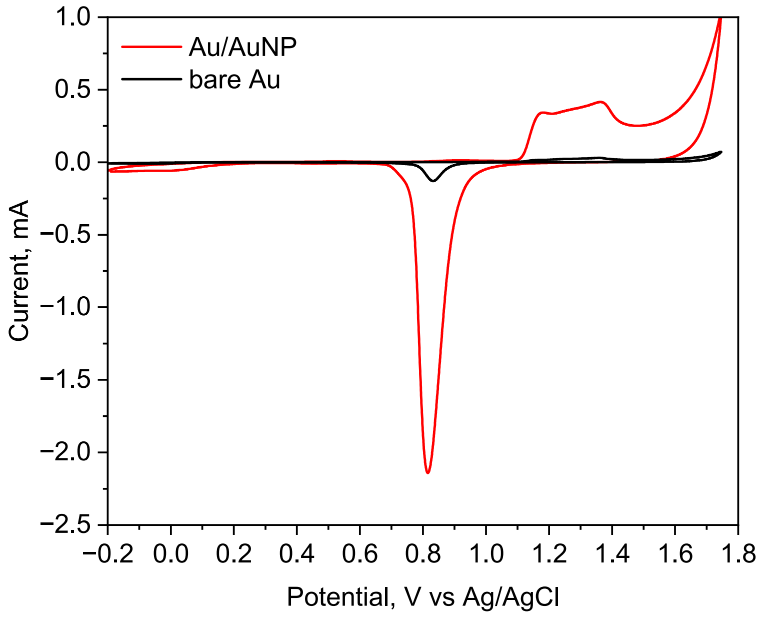

Figure S1. Cyclic voltammetry (CV) experiment during the electrochemical cleaning of a bare gold electrode (a) and an electrode with gold nanoparticles (b) in 0.5 M H_2SO_4, sweep ratio is 200 mV/s

Above 1.2 V versus Ag/AgCl, the gold on the electrode surface oxidizes to gold ions (Au^{3+}), leading to an anodic current in these CVs (Figure S1). These Au^{3+} ions are subsequently reduced at 0.82 V, where corresponding cathodic currents are observed. We approximate these cathodic current bands as Gaussian curves. This approximation allows us to assume that the ratio of their intensities is directly proportional to the ratio of their areas. Therefore, we calculated the increase in the electrochemical area of the electrode after the deposition of nanoparticles using the ratio of these cathodic currents: $I_{Au/AuNp}/I_{Au}$.

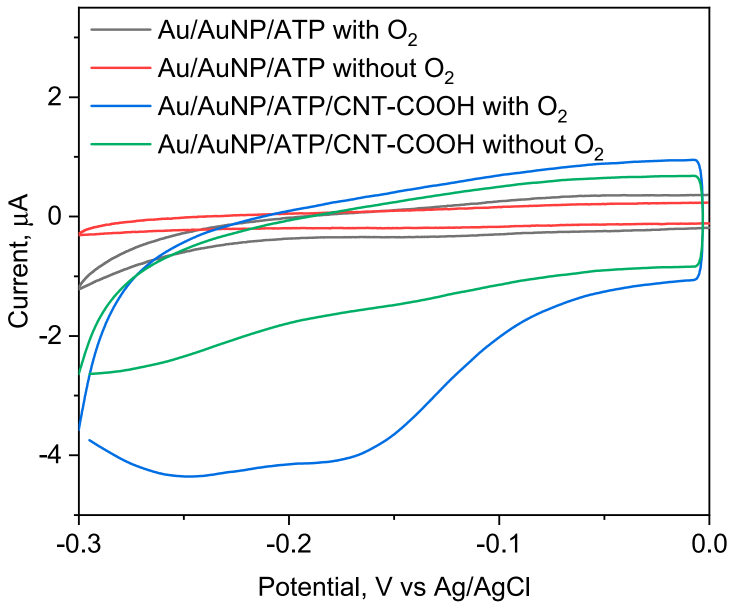

Figure S2. Cyclic voltammetry (CV) experiment of Au/AuNP/CNT-COOH with ambient molecular oxygen and without dioxigen after bubbling with argon for 5 min in a solution of 50 mM PPB and 100 mM KCl, sweep ratio is 20 mV/s

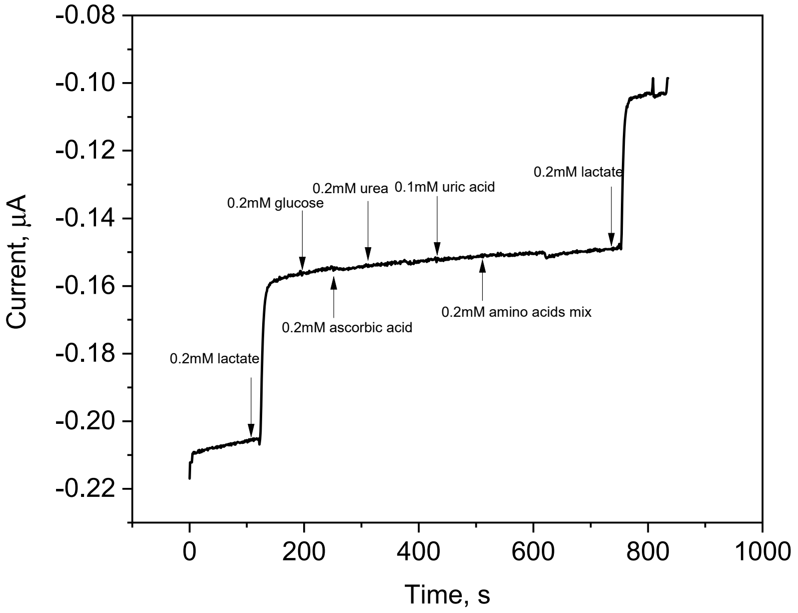

Figure S3. Test of interferences of the Au/AuNP/CNT-COOH/LOx+CAT electrode using chronoamperometry at −0.20 V vs Ag/AgCl. The experiment started with 0.2 mM L-lactate and

then added D-glucose (0.2 mM), L-ascorbic acid (0.2 mM), urea (0.2 mM), uric acid (0.1 mM), a mixture of amino acids (0.2 mM) as interferences, and at the end, L-lactate (0.2 mM)

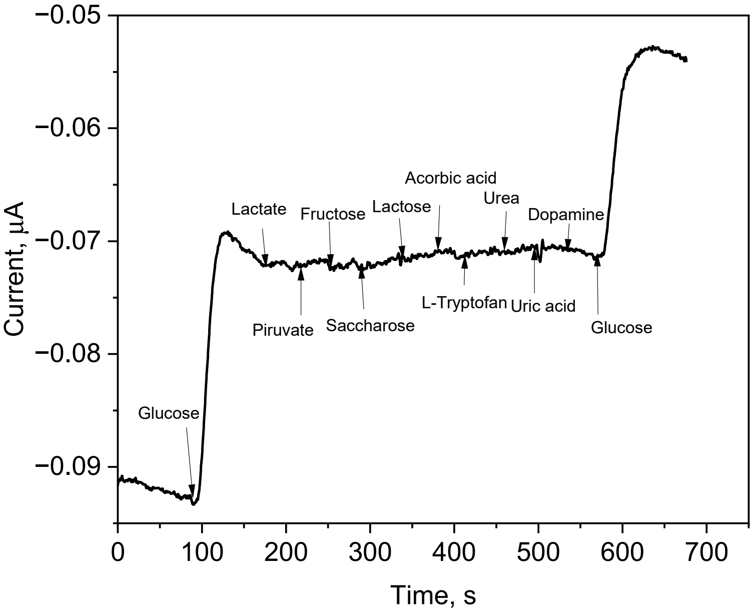

Figure S4. Test of interferences of the Au/AuNP/CNT-COOH/GOx+CAT electrode using chronoamperometry at −0.20 V vs Ag/AgCl: the experiment started with 1 mM glucose and then added lactate (0.5 mM), piruvate (0.5 mM), fructose (0.5 mM), saccharose (0.5 mM), lactose (0.5 mM), ascorbic acid (0.1 mM), tryptofan (0.1 mM), urea (0.5 mM), uric acid (0.1 mM), dopamine (50 µM) as interferences, and at the end was added again glucose (1 mM)

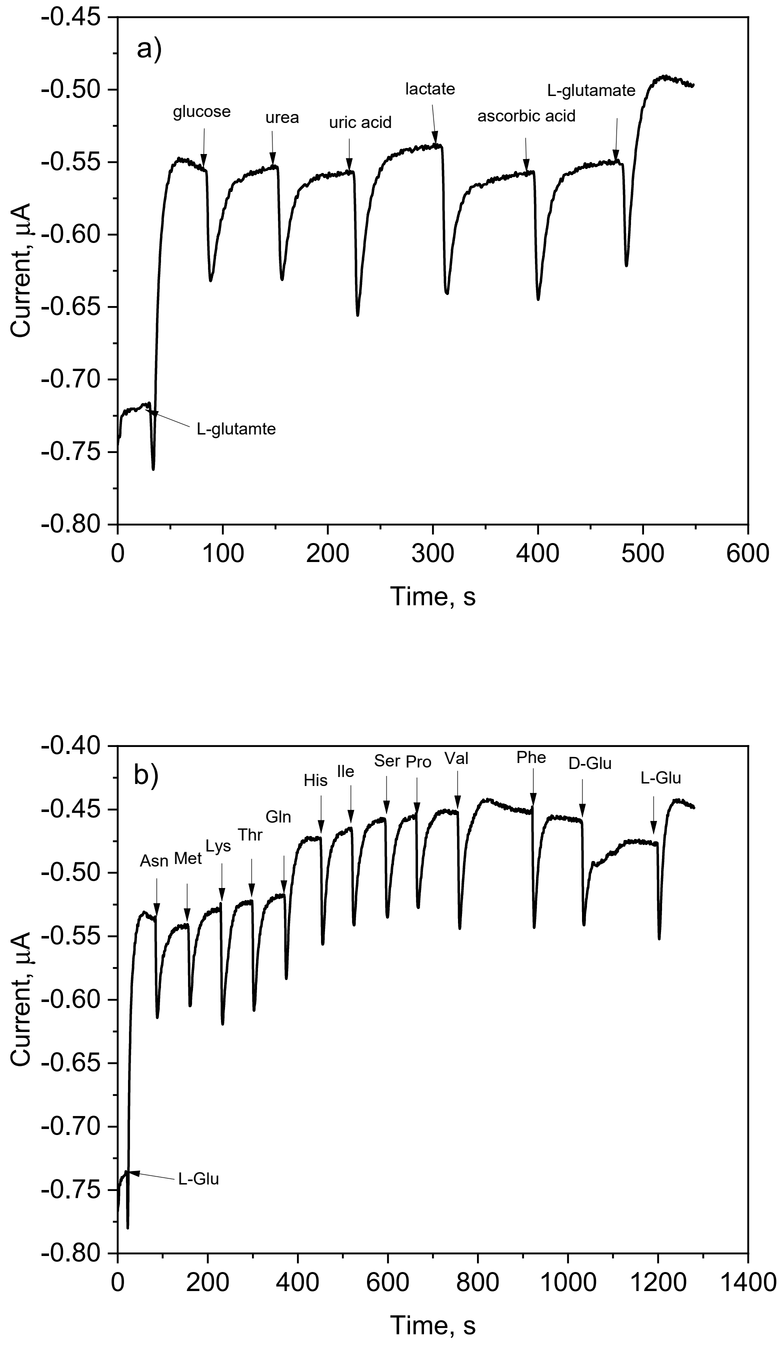

Figure S5. Test of interferences of the Au/AuNP/CNT-COOH/LGOx+CAT electrode using chronoamperometry at −0.20 V vs Ag/AgCl: a) the experiment started with 1 mM L-glutamate and then added D-glucose (1 mM), L-ascorbic acid (1 mM), urea (1 mM), uric acid (0.1 mM) as interferences, and at the end was added again L-glutamate (1 mM); b) the test of most common L-amino acids (Asn, Met, Lys, Thr, Gln, His, Ile, Ser, Pro, Val, Phe) and D-glutamate of 1 mM

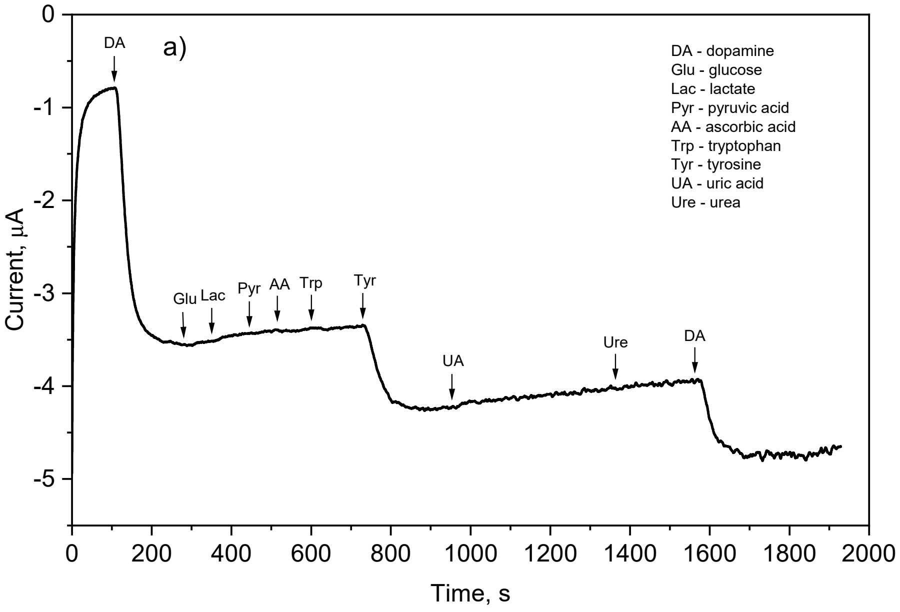

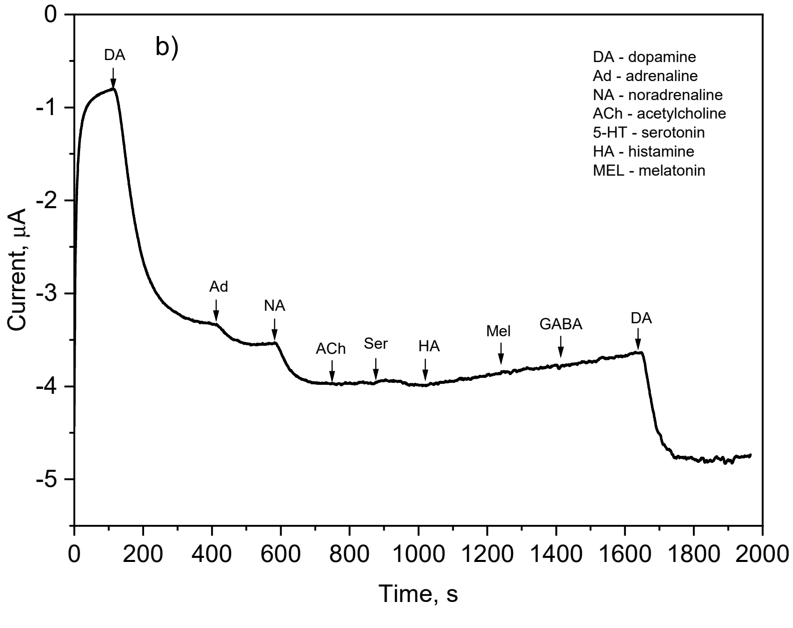

Figure S6. Test of interferences of the Au/AuNP/CNT-COOH/TYR bioelectrode using chronoamperometry at −0.20 V vs Ag/AgCl: a) the test of the most common interferences and aromatic amino acids (Tyr and Trp); b) the test of neuromediators. All concentrations of the reagents were 50 µM

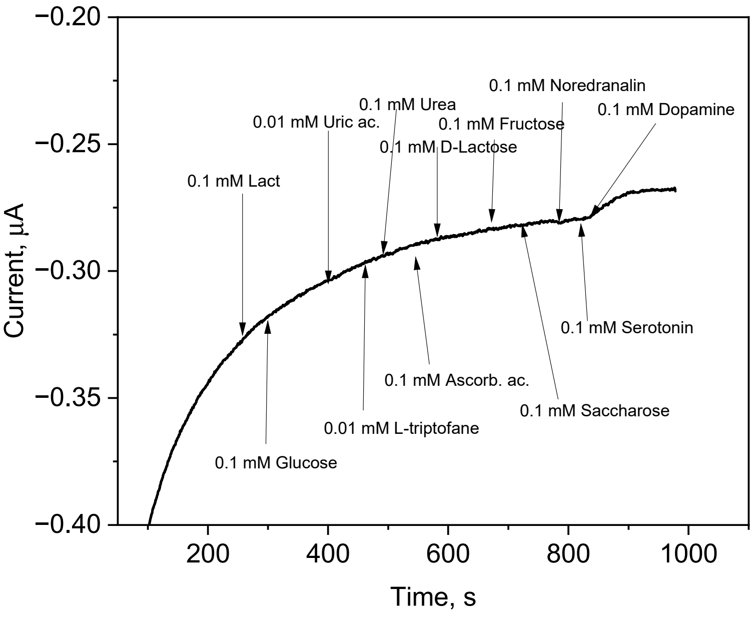

Figure S7. Test of interferences of the Au/AuNP/CNT-COOH electrode using chronoamperometry at −0.20 V vs Ag/AgCl

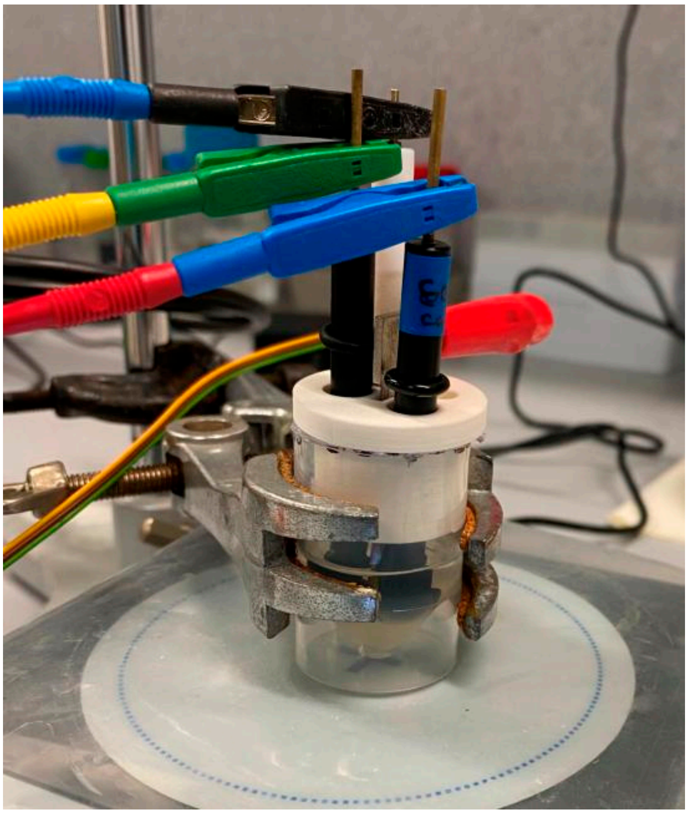

Figure S8. Electrochemical cell for the analysis of real samples

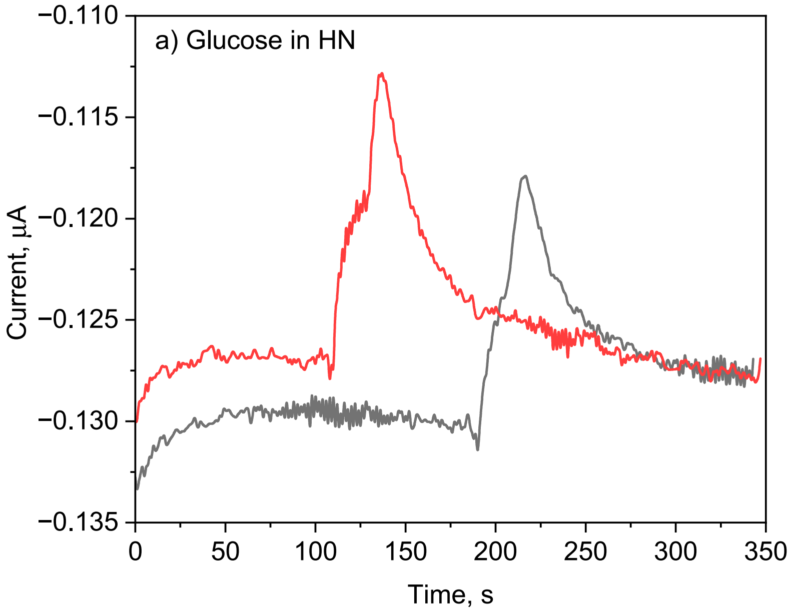

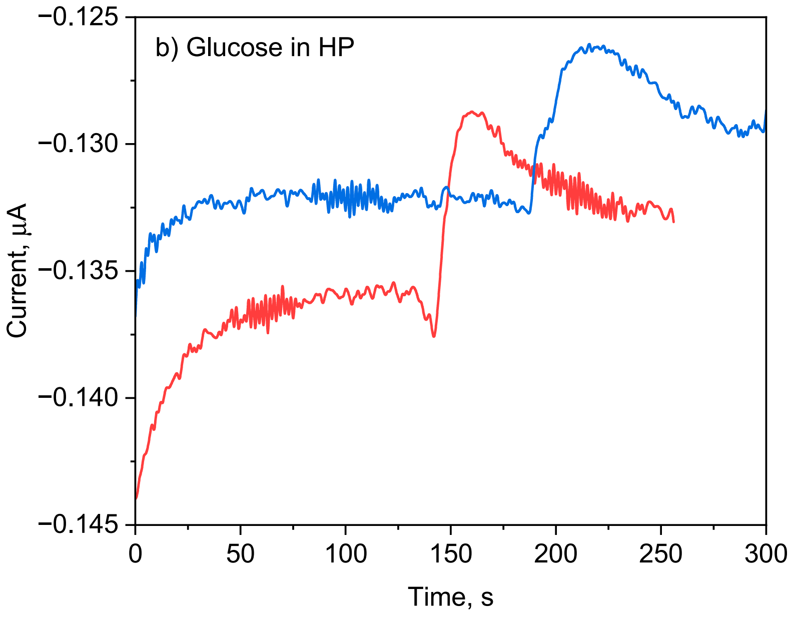
b) Glucose in HP
−0.125
−0.130
−0.135
−0.140
−0.145
Current, µA
0
50
100
150
200
250
300
Time, s

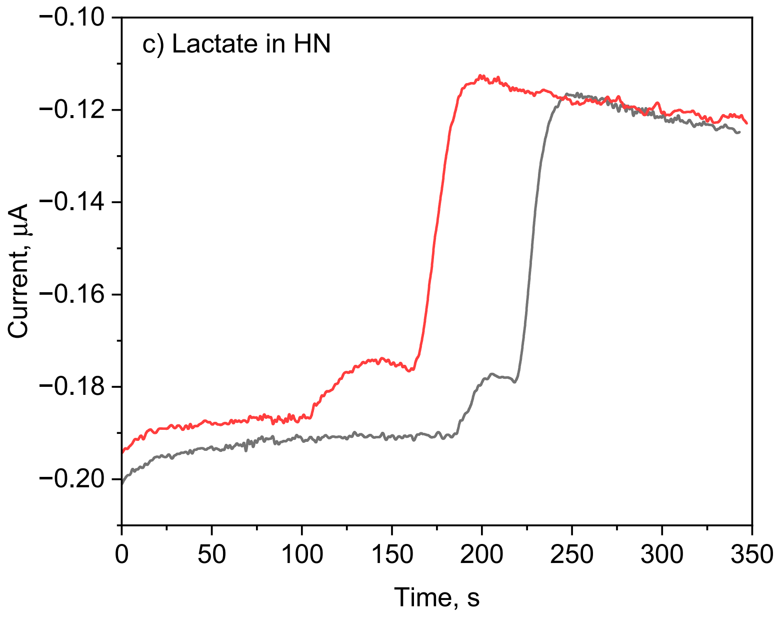
c) Lactate in HN
−0.10
−0.12
−0.14
−0.16
−0.18
−0.20
Current, µA
0
50
100
150
200
250
300
350
Time, s

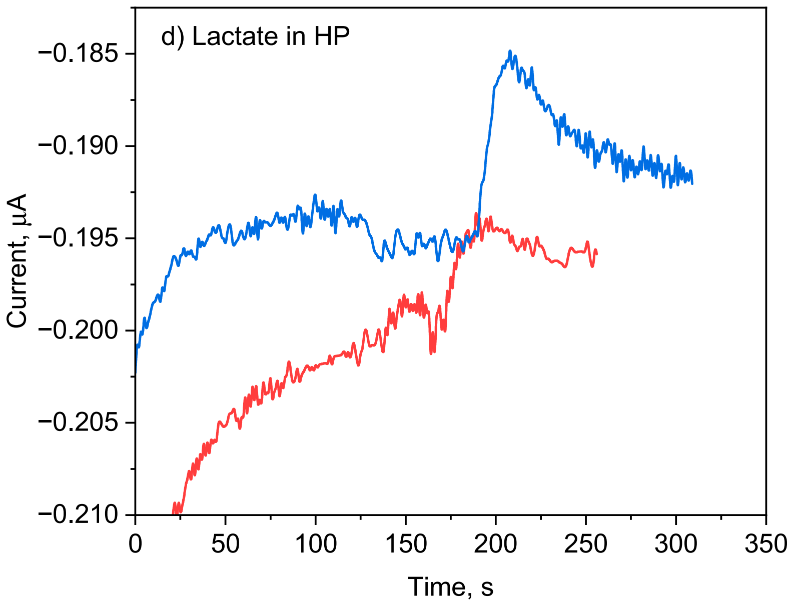

Figure S9. Chronoamperometry curves during the analysis of glucose (a and b), lactate (c and d) in the HN (a and c) and HP (b and d) serums in PPB and at the potential of −0.20 V versus Ag/AgCl. The different colors of the traces in each figure represent different sample loading.
